# Post‐operative septic arthritis after anterior cruciate ligament reconstruction results in higher osteoarthritis severity and deficit of range of motion: A matched control analysis with a minimum 8‐year follow‐up

**DOI:** 10.1002/jeo2.70716

**Published:** 2026-04-14

**Authors:** Sebastian Imach, Steffen T. Ubl, Carolin Flieser, Julius Stüber, Sven Shafizadeh, Arasch Wafaisade, Jan‐Hendrik Naendrup, Daniel Guenther, Bertil Bouillon, Thomas R. Pfeiffer

**Affiliations:** ^1^ Department of Orthopaedic Surgery, Trauma Surgery and Sports Medicine, Cologne Merheim Medical Center Witten/Herdecke University Cologne Germany; ^2^ Department of Orthopedic Surgery and Sports Traumatology Sana Medical Centre Cologne Germany

**Keywords:** infection after ACL reconstruction, knee joint complications, long‐term clinical outcomes, osteoarthritis severity, range of motion limitation

## Abstract

**Purpose:**

Evaluating knee joint function, activity level, and osteoarthritis severity in patients at least 8 years after post‐operative knee septic arthritis (SA) following anterior cruciate ligament reconstruction (ACLR).

**Methods:**

From May 2010 to January 2012, 39 patients at our institution were treated for knee SA following ACLR using graft‐retaining treatment protocols. Follow‐up examinations after a minimum of 8 years included clinical examination, measurement of anterior tibial translation (rolimeter), International Knee Documentation Committee Subjective Knee Form (IKDC), 12‐Item Short Form Health Survey (SF‐12), Western Ontario and McMaster Universities Osteoarthritis Index (WOMAC), and Marx and Tegner scores. Osteoarthritis severity was described on radiographs using the Kellgren–Lawrence scale. Synovial fluid was aspirated from patients with persistent infection signs and evaluated using multiplex polymerase chain reaction (PCR). Based on ACLR graft, sex and preoperative Tegner score (±1), a 1:1 propensity score matched control group including patients with ACLR without knee SA signs was assembled.

**Results:**

Matching resulted in 17 patients per group. While the patient‐reported outcome measures (PROMs) (IKDC, Tegner, Marx and SF‐12) showed no significant differences between the groups (*p* > 0.05), the WOMAC score was significantly worse in the infection group (*p* = 0.016). Range of motion deficits were more frequent in the infection group (65% vs. 18%; *p* = 0.005). The infection group also had higher Kellgren–Lawrence grades (2 [1–2.75] vs. 0 [0–1], *p* < 0.001). Multiplex PCR detected no persistent infection. Two patients (10%) in the infection group required graft removal. No correlation was found between the number of lavages and long‐term outcomes.

**Conclusions:**

SA after ACLR, when treated with a standardized graft‐retaining protocol, results in higher OA severity, worse WOMAC scores and persistent range of motion limitations at long‐term follow‐up, while other PROMs and activity level remained comparable to those of non‐infected cases.

**Level of Evidence:**

Level III.

**Abbreviations**
ACL: anterior cruciate ligament
ACLR: anterior cruciate ligament reconstruction
BMI: body mass index
IKDC: International Knee Documentation Committee Subjective Knee Form
IQR: interquartile range
KL: Kellgren–Lawrence
MCID: minimal clinically important difference
MOON: Multicenter Orthopaedic Outcomes Network
OA: osteoarthritis
PCR: polymerase chain reaction
PROMs: patient‐reported outcome measures
ROM: range of motion
SA: septic arthritis
SD: standard deviation
SF‐12: 12‐Item Short Form Health Survey
WOMAC: Western Ontario and McMaster Universities Osteoarthritis Index

## INTRODUCTION

Post‐operative septic knee arthritis (SA) is a rare but feared complication after anterior cruciate ligament reconstruction (ACLR). SA incidence following ACLR without vancomycin soaking ranges between 0.14% and 1.8% [3, 7].

SA following ACLR is a risk factor for knee instability and osteoarthritis (OA) [6, 13]. In the past, early graft removal was the preferred treatment option for infection, which is associated with greater flexion and extension deficits and lower Lysholm scores [9, 13, 21]. After graft removal, staged revision ACLR was an option to restore knee stability [25].

Current standardized protocols for the management of SA following ACLR consist of graft‐preserving arthroscopic management with recurrent lavages and targeted antibiotic therapy for up to 6 weeks [2, 13, 16, 22]. Avoiding delayed diagnosis is essential to reduce the likelihood of graft removal and restricted range of motion (ROM) [24]. Moreover, Lo Presti et al. reported eradication of the infection after a single arthroscopic debridement procedure with extra‐articular hardware removal, and manual and arthrometric testing at 1‐year follow‐up showed no abnormal findings [12].

After a mean follow‐up of 48 months, signs of early‐stage OA on magnetic resonance imaging were present in patients with SA [15]. However, the mid‐term outcomes of an arthroscopic treatment protocol demonstrated good‐to‐excellent clinical results after a follow‐up of 4.7 years with graft retention in 97.2% of all cases [22]. In this study, patients with initial graft retention had significantly higher subjective functional outcomes and higher return to sports rate scores [19, 23]. At our institution, we demonstrated successful initial infection management with graft preservation in 37 out of 41 cases between 2010 and 2012 by a standardized protocol over a short‐term follow‐up of 10 ± 7 months [16]. Long‐term follow‐up data are still missing, especially regarding long‐lasting pathogen eradication and the development of OA as defined by radiological images.

Therefore, this study aimed to evaluate knee joint function, activity level, and OA severity in patients with post‐operative SA following ACLR after a minimum follow‐up of 8 years who were treated with a standardized, graft‐retaining protocol. It was hypothesized that functional scores, activity level, laxity testing and OA severity would be significantly worse than those in a matched control group following ACLR without any signs of post‐operative SA.

## METHODS

### Study population

After approval by the local ethics committee (University of Witten/Herdecke, No. 203/2018), we retrospectively identified all patients treated for early post‐operative knee SA within 4 weeks after primary ACLR between May 2010 and January 2012 at our specialized joint surgery centre. Inclusion criteria were defined as increasing pain, swelling, limitation of knee function and at least one common sign of infection (temperature > 38°C, shivering and malaise) after isolated ACLR with or without meniscal surgery. All patients underwent arthrocentesis and were included if the synovial white blood cell count exceeded 50,000 cells µL^−1^ [19]. Positive microbiological cultures were not required for inclusion. Demographic data, surgical details of the index ACLR, microbial detection, graft retention or removal and initial Gächter classification [5] were obtained by reviewing the medical records and operative reports. A control group of patients who underwent ACLR with or without meniscal surgery at our institution during the same period, without evidence of post‐operative SA, was established. Controls were selected and matched to the infection group based on predefined criteria, and details of the matching procedure are provided in the “Statistical analysis” section. All ACL grafts were fixed using suspensory fixation on the femoral side and hybrid fixation on the tibial side. A standard perioperative antibiotic prophylaxis consisting of a first‐ or second‐generation cephalosporin administered intravenously was given prior to primary ACLR.

### Treatment protocol

All patients in the infection group underwent a standardized treatment protocol of at least three arthroscopic lavages and partial synovectomies of all knee compartments through three incisions (anterolateral, anteromedial and posteromedial portal) by a single senior surgeon (S.S.) within 24 h of admission. No perioperative antibiotic prophylaxis was administered during the first surgical procedure. The procedure was repeated at 2‐day intervals until there was no evidence of SA during surgery. A graft retention protocol was used in all cases, and intra‐articular closed‐suction drainage was applied after each surgical procedure. The drain remained in situ between procedures and was removed 2 days after the final lavage when drainage output was below 50 mL per 24 h. Empiric antibiotic therapy with coverage for gram‐positive bacteria at the recommended dosage was initiated immediately after the first lavage, and renal function, assessed by glomerular filtration rate, was monitored every 2 days, with dosing adjusted accordingly. The VITEK® 2 system was used for pathogen identification, as well as resistance testing, and cultures were incubated in the Bactec System® (Co. Becton Dickinson) for 14 days. As soon as microbiological data were available, pathogen‐specific, susceptibility‐guided antibiotic therapy was administered intravenously for at least 10 days, followed by an oral regimen to complete a total treatment duration of 6 weeks. In addition, all patients received nonsteroidal anti‐inflammatory drugs. After complete remission of the infection, full ROM was allowed with continuous passive motion therapy and partial weight bearing at 20% of the patient's body weight for 6 weeks.

### Follow‐up examination

All eligible patients were contacted and invited for follow‐up examinations at a minimum of 8 years. The clinical examination consisted of measurement of anterior tibial translation using a Rolimeter (Aircast, Europe) during a Lachman‐type manoeuvre at 25° of knee flexion, passive ROM testing in the supine position of each knee with a standard goniometer, and determination of the percentage of side‐to‐side muscle deficit measured 10 and 20 cm above the patella, at the level of the patella, and 15 cm below the patella. Thresholds for extension and flexion deficits were defined as side‐to‐side differences of 5° and 10°, respectively. Patient‐reported outcome measures (PROMs) included the Tegner Activity Scale, Marx Activity Rating Scale, International Knee Documentation Committee Subjective Knee Form (IKDC), 12‐Item Short Form Health Survey (SF‐12) and Western Ontario and McMaster Universities Osteoarthritis Index (WOMAC). OA severity was assessed on standing radiographs of both knees (Rosenberg view) using the Kellgren–Lawrence (KL) scale by two blinded senior orthopaedic surgeons (T.R.P. and S.S.).

Patients with the aforementioned signs of persistent infection at the time of follow‐up underwent needle aspiration of the synovial fluid and were evaluated for cell count, leucocyte differentiation, microbiological culture and multiplex polymerase chain reaction (PCR). A PCR sample was considered positive if DNA from the initial pathogen diagnosed during the surgical management of the infection was detected.

### Statistical analysis

Statistical analyses were performed using the R software Version 4.3.2 (R Foundation for Statistical Computing). A 1:1 propensity score matching was performed using nearest‐neighbour matching with a calliper width of 0.2 times the standard deviation (SD) of the logit of the propensity score of the infection and control groups. Covariates included were ACL graft (hamstring or quadriceps tendon with or without bone block autograft), sex and preoperative Tegner score (±1).

Descriptive statistics are presented as mean ± SD for normally distributed variables or median (interquartile range [IQR]) for non‐normally distributed or ordinal variables, with normality assessed using the Shapiro–Wilk test. Differences between groups were tested using the Wilcoxon rank‐sum test, Pearson's Chi‐square test and Fisher's exact test. Correlations were evaluated using Spearman's rank correlation. Observer agreement was assessed with weighted Cohen's kappa (*κ*) and interpreted as almost perfect (*κ* > 0.80), substantial (*κ* = 0.61–0.80), moderate (*κ* = 0.41–0.60), fair (*κ* = 0.21–0.40) or poor (*κ* < 0.21) [11]. Statistical significance was set at *p* < 0.05.

## RESULTS

### Demographic and anthropometric characteristics

In the infection group, 20 out of 39 eligible patients (51%) were available for follow‐up examination. Table 1 shows a comparison of the demographic characteristics between patients lost to follow‐up and those with available follow‐up.

**Table 1:** Demographic and anthropometric data of patients lost to follow‐up compared with patients with available follow‐up.

	Follow‐up available (*n* = 20)	Lost to follow‐up (*n* = 19)	*p*
Age at surgery (years)	35.0 [27.5–42.3]	27.0 [18.0–30.0]	0.016
Sex			0.044
Female	6 (30%)	1 (5%)	
Male	14 (70%)	18 (95%)	
BMI (kg/m^2^)	25.2 [23.8–30.0]	24.0 [22.6–28.4]	n.s.
Meniscal surgery	8 (40%)	8 (42%)	n.s.
Repair	5	3	
Partial meniscectomy	3	5	
ACL graft			n.s.
Hamstring tendon	18 (90%)	19 (100%)	
Quadriceps tendon	2 (10%)	0	

Abbreviations: ACL, anterior cruciate ligament; BMI, body mass index.

In the infection group, a pathogen was initially detected in 17 (85%) patients, with Staphylococcal species identified in 16 (94%) cases and *Cutibacterium acnes* identified in one (6%) patient. No pathogen was identified in three patients (15%). In the infection group, graft resection and bone grafting due to graft failure were performed in two (10%) patients who later declined revision ACLR due to sequelae. Only one patient in the infection group underwent revision surgery (meniscal surgery) during the subsequent period. In the control group, four revision surgeries were performed on the involved knee in three patients, with two ACL failures receiving ACL revision and two partial meniscus resections. In both groups, contralateral ACL tears occurred in two patients.

Propensity score matching yielded 17 pairs. The median follow‐up duration was 8.8 years [8.2–9.4] in the infection group and 10.0 years [9.8–10.1] in the control group. The demographic characteristics of the patients are shown in Table 2.

**Table 2:** Demographic and anthropometric data of propensity score‐matched patients for ACL graft, sex and preoperative Tegner score (±1).

	Infection group (*n* = 17)	Control group (*n* = 17)	*p*
Age at surgery (years)	35.0 [27.0–40.0]	37.0 [26.0–42.0]	n.s.
Sex			n.s.
Female	3 (18%)	3 (18%)	
Male	14 (82%)	14 (82%)	
BMI (kg/m^2^)	25.2 [23.8–26.6]	23.6 [22.2–29.3]	n.s.
Tegner score	8 [5–9]	8 [5–9]	n.s.
Meniscal surgery	7 (41%)	11 (65%)	n.s.
Repair	3	0	
Partial meniscectomy	4	11	
ACL graft			n.s.
Hamstring tendon	15 (88%)	15 (88%)	
Quadriceps tendon	2 (12%)	2 (12%)	
Gächter stage			
1	12 (71%)		
2	4 (23%)		
3	1 (6%)		

*Note*: Values given as median [IQR] for continuous and ordinal variables and n (%) for categorical variables.

Abbreviations: ACL, anterior cruciate ligament; BMI, body mass index; n.s., not significant.

### PROMs

The total WOMAC score was significantly higher (i.e., worse) in the infection group (*p* = 0.016). In the evaluation of subcategories, the two groups differed significantly in the function subcategory (*p* = 0.031), but not in pain or stiffness (*p* > 0.05). No significant differences were observed between the groups at follow‐up for the Tegner score, post‐operative reduction in Tegner score, Marx score, IKDC score or SF‐12 scores (*p* > 0.05). The PROMs values are listed in Table 3.

**Table 3:** Subjective outcome scores.

	Septic knee arthritis (*n* = 17)	Control group (*n* = 17)	*p*
Tegner	5 [4–6]	6 [4–8]	n.s.
Tegner reduction	−2.0 [−4.0 to 0.0]	0.0 [−1.0 to 0.0]	n.s.
Marx	6 [1–8]	7 [4–8]	n.s.
IKDC	80.5 [60.9–83.9]	88.5 [66.7–93.1]	n.s.
SF12 Physical	52.8 [45.1–55.9]	54.5 [53.7–56.2]	n.s.
SF12 Mental	57.1 [43.1–59.3]	57.8 [2.8–58.8]	n.s.
WOMAC	7 [3–12]	2 [0–3]	0.016
Pain	1 [0.5–4]	0 [0–1]	n.s.
Stiffness	1 [0.5–2.5]	0 [0–2]	n.s.
Function	4 [1–7]	0 [0–2]	0.031

*Note*: Variables are reported as a median [IQR]. Level of significance: 05.

Abbreviations: IKDC, International Knee Documentation Committee Subjective Knee Form (0–100; higher scores indicate better knee function); IQR, interquartile range; Marx, Marx Activity Rating Scale (0–16; higher scores indicate higher activity level); n.s., not significant; SF‐12, 12‐Item Short Form Health Survey (0–100; higher scores indicate better health status); Tegner, Tegner Activity Scale (0–10; higher scores indicate higher activity level); WOMAC, Western Ontario and McMaster Universities Osteoarthritis Index (0–100; higher scores indicate worse symptoms and functional limitation.

### Clinical examination

**Figure 1:**
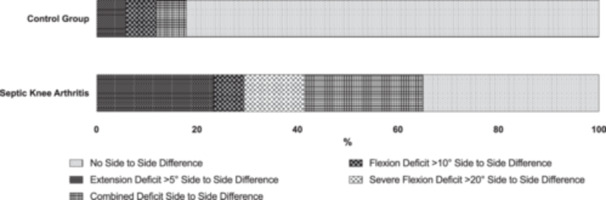
Distribution of side‐to‐side knee range‐of‐motion deficits in the control group (top) versus the septic knee arthritis group (bottom). The stacked bars indicate the proportion of individuals with no difference, flexion deficit >10°, severe flexion deficit >20°, extension deficit >5° or combined deficits.

In the infection group, a significantly higher number of patients showed restrictions in ROM compared to the control group (65% vs. 18%, respectively) (*p* = 0.005) (Figure 1). Among these patients, four had an extension deficit compared to one in the control group, with a mean restriction of 7.5 ± 5.5° and 5.0°, respectively. Additionally, three patients in the infection group had a flexion deficit compared with one in the control group, with flexion inhibition of 17.6 ± 7.0° (range = 10–28°) and 10.0°, respectively. In the infection group, four patients had both flexion and extension deficits, whereas only one of the patients in the control group had these combined deficits. There were no significant differences between the two groups in terms of side‐to‐side differences in anterior tibial translation (infection group: 1.4 ± 2.3 mm; control group: 1.1 ± 3.1 mm, *p* > 0.05).

### Radiographic outcome

**Figure 2:**
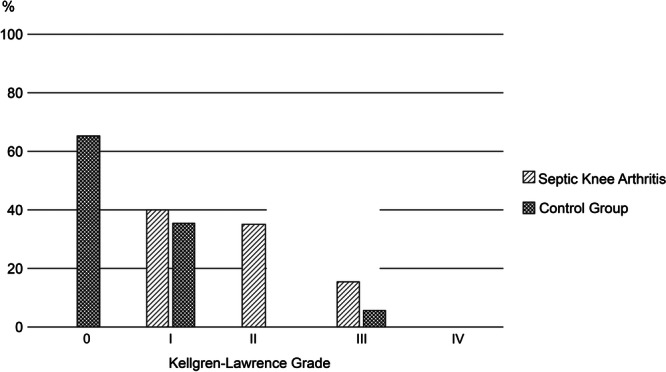
Radiographic distribution of Kellgren–Lawrence grades in the septic knee arthritis group (grey‐shaded bars) compared with the control group (dark hatched bars). The *y*‐axis indicates the percentage of participants classified within each radiographic grade. The affected knee in the infection group demonstrated significantly higher Kellgren–Lawrence grades compared with the control group (2 [IQR 1–2.75] vs. 0 [IQR 0–1]; *p* < 0.001). IQR, interquartile range.

The affected knee joint in the infection group had a significantly higher KL grade compared to the affected knee of the control group (2 [1–2.75] vs. 0 [0–1]; *p* < 0.001) (Figure 2). The comparison of the difference between the affected and unaffected sides of the infected and the control group differed significantly (1.5 [0.25–2.0] vs. 0 [0–0.5]; *p* = 0.04). Regarding interobserver reliability, the KL grading showed moderate repeatability (*κ* = 0.60).

No treatment‐related complications were observed during hospitalization. At follow‐up, three patients underwent synovial fluid aspiration. However, neither microbiological examination nor PCR of the synovial aspirate revealed persistent SA in any of the patients. The mean cell count in the synovial analysis was 83/µL in these cases.

The initial Gächter stage was found to be significantly correlated with the degree of flexion inhibition (*r* = 0.457; *p* = 0.03) and WOMAC score (*r* = 0.579; *p* = 0.008) but not with extension inhibition, anterior tibial translation, IKDC, Marx, Tegner score or KL grades. No correlation was found between the number of lavages performed (median = 3, range = 3–7) and the long‐term outcomes.

## DISCUSSION

The main finding of this study was that SA following ACLR, when managed with graft retention, resulted in a significantly higher incidence of restricted ROM, higher OA severity and worse WOMAC scores, while other PROMs and activity level remained comparable to those in a matched control cohort without evidence of post‐operative SA.

Management of SA, its impact on subjective and functional outcomes, and the return to activity are topics of great interest because of their devastating impact on knee health. A recent study of 38 patients with post‐operative SA after ACLR, with a mean follow‐up of 5 years and a minimum follow‐up of 2 years, showed a high return to sports rate, but predominantly at a reduced frequency [23]. Another retrospective study of 27 patients found reduced PROMs compared with a matched group with a mean follow‐up of 5 years, ranging from 13 to 108 months [2].

Our study did not find any differences in the PROMs between the groups. The only statistically significant difference in the total WOMAC score was the worst outcome in the infection group. However, the results did not exceed the established minimal clinically important difference (MCID) as defined for OA of the lower extremities [14]. This contrasts with the findings of previous studies. For example, at a mean follow‐up of 5 years, patients with post‐operative SA after ACLR showed a reduced Tegner activity score compared to a matched control group [1, 2]. This discrepancy may be related to differences in follow‐up duration between studies. However, as midterm post‐operative data were not available in the present study, no conclusions can be drawn regarding the temporal development of activity levels or PROMs. Furthermore, no differences were found between the two groups in terms of side‐to‐side differences in anterior tibial translation, which is consistent with previous short‐ and mid‐term follow‐up studies showing good knee stability after SA following ACLR [12, 22]. This further supports the results of previous studies in which persistent ACL deficiency was the only patient‐dependent factor significantly associated with a reduction in sports activity [18, 23], highlighting the importance of graft retention in the event of infection following ACLR, whenever clinically feasible. Furthermore, early detection of SA is paramount, as the WOMAC score correlated with the initial Gächter stage found intraoperatively in this study.

A major concern after ACLR is persistent stiffness and arthrofibrosis, which are the main causes of limited knee function [17, 23]. The present study found a significantly higher number of patients with either a flexion or extension deficit and a high prevalence of combined ROM deficits in patients with infection after ACLR. This is consistent with a previous study of 24 infection cases with a mean follow‐up of 5 years, which found an extension deficit >5° in 21% of patients and a flexion deficit >10° in 21% [1]. Regarding the prevention of ROM deficits, the early detection of SA is once again crucial, as the degree of flexion limitation is correlated with the initial Gächter stage. This is consistent with a previous study, which showed that delayed diagnosis after 7 days could result in a limited ROM, but also necessitated a longer course of antibiotic therapy and increased the likelihood of graft removal [24].

**Figure 3:**
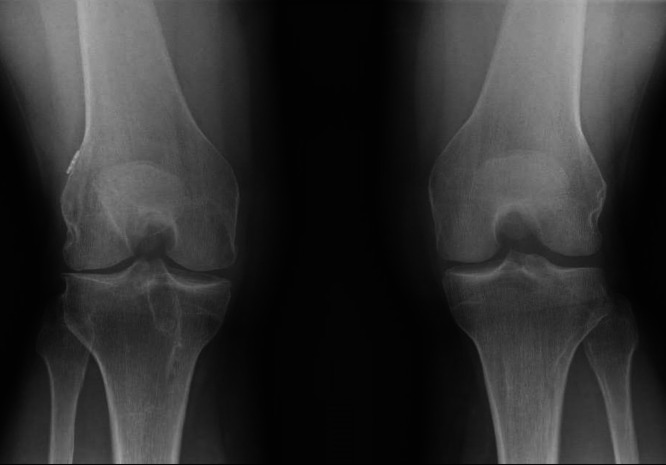
Rosenberg view of both knees of a 44‐year‐old female patient (body mass index = 22.9 kg/m^2^) at a follow‐up of 106 months after post‐operative septic knee arthritis (SA) following anterior cruciate ligament reconstruction (ACLR). The right knee demonstrates status post ACLR using an ipsilateral hamstring tendon autograft, complicated by post‐operative SA caused by *Staphylococcus epidermidis*. A graft‐retaining treatment protocol with three arthroscopic lavages was performed. The affected knee exhibits a persistent 10° extension deficit and advanced osteoarthritic changes (Kellgren–Lawrence grade III) compared with the contralateral knee (Grade I), with no clinically relevant side‐to‐side anterior instability (4 mm vs. 2 mm).

SA can also cause articular cartilage damage [18]. A previous study of 27 patients with SA after ACLR found evidence of subtle cartilage degeneration; however, it could not demonstrate differences from a matched control group at 5 years of follow‐up [2]. These results are supported by another mid‐term follow‐up study of 26 patients in which worsening OA was observed only after extensive meniscal resection [20]. However, a study of four patients with a long‐term follow‐up of 17.9 years showed that all patients showed progression of cartilage loss [20]. This trend of ongoing cartilage degeneration and progression of OA in the long term is further supported by the present study. Significantly higher KL grades were found compared to the control group and the contralateral unaffected side (Figure 3). Notably, the WOMAC score, which assesses the symptoms of knee OA, was significantly different between the two groups. However, the differences did not exceed the MCID for the WOMAC score.

Despite that, progression of KL grades of one grade (73%) or two grades (23%) has been shown by the MOON group in young active patients even after regular ACLR without post‐operative SA at 10 years of follow‐up [4]. Furthermore, the prevalence of knee OA after ACLR increases significantly in the second decade after surgery. Thus, further progression of OA can be expected in the infection group [8, 14], emphasizing the importance of prevention and a standardized treatment protocol in the event of infection.

The European Bone and Joint Infection Society and the European Society for Sports Traumatology, Knee Surgery, and Arthroscopy have developed specific recommendations regarding treatment strategies for knee joint infections. Arthroscopic debridement with graft retention combined with 6 weeks of antibiotic therapy is recommended as soon as infection is clinically suspected. Additional debridement was indicated if the clinical course was unfavourable and signs of infection persisted, with the Gächter classification and graft status being the main determinants of the decision to retain or remove the graft [17].

This study successfully demonstrated the potential of a standardized treatment protocol for the long‐term eradication of pathogens during long‐term follow‐up. The result of no late reoperations due to persistent infection meets the expectations of patients who prefer a more extensive initial treatment to avoid late reoperations [10]. Accordingly, the two patients who required graft resection did not opt for a new ACLR, which is consistent with a previous study in which eight of twelve patients who required graft removal did not undergo revision ACLR [18].

### Limitations

This study has some limitations. First, owing to the retrospective nature of this study, confounding and selection biases cannot be ruled out. Second, the higher number of partial meniscal resections in the control group may have influenced OA severity and should be considered when interpreting group comparisons. Third, despite considerable efforts, the patient follow‐up rate was low (51%). This limitation reduces the statistical power of the study and increases the risk of Type II error. However, as seen in previous studies, the incidence of joint infection after ACLR is low, and the number of patients is comparable. Importantly, significant differences in age and sex between patients with available follow‐up and those lost to follow‐up suggest potential attrition‐related selection bias, which may limit the generalizability and external validity of our findings. Fourth, ACL laxity was assessed using a Rolimeter (Aircast, Europe), which may be user‐dependent. Finally, given that the study population was partially composed of tertiary referrals, the incidence of post‐operative infection following ACLR cannot be estimated from this cohort.

## CONCLUSIONS

SA after ACLR, when treated with a standardized graft‐retaining protocol, results in higher OA severity, worse WOMAC scores, and persistent ROM limitations at long‐term follow‐up, while other PROMs and activity level remained comparable to those of non‐infected cases.

## AUTHOR CONTRIBUTIONS

All authors contributed to the study's conception and design. Material preparation and data collection were performed by Sebastian Imach, Steffen T. Ubl, Julius Stüber, Sven Shafizadeh and Jan‐Hendrik Naendrup. Measurements were performed by Steffen T. Ubl, Sebastian Imach and Carolin Flieser. The first draft of the manuscript was written by Carolin Flieser, Sebastian Imach and Steffen T. Ubl, Thomas R. Pfeiffer; Sven Shafizadeh, Bertil Bouillon, Daniel Guenther and Arasch Wafaisade made meaningful corrections to the structure of the article and guided the statistical methods and data processing. All authors commented on the previous versions of the manuscript. All the authors have read and approved the manuscript.

## CONFLICT OF INTEREST STATEMENT

Thomas R. Pfeiffer receives research grants from Arthrex outside the submitted work. The remaining authors declare no conflicts of interest.

## ETHICS STATEMENT

Approval was obtained from the University of Witten/Herdecke (Nr. 203/2018).

## Data Availability

The data sets utilized during the current study are available from the corresponding author on reasonable request.
